# Long‐Term Temporal Profile of Motor Recovery After Intracerebral Hemorrhage

**DOI:** 10.1002/acn3.70124

**Published:** 2025-08-05

**Authors:** Yan Zheng, Fu‐Xin Lin, Ling‐Yun Zhuo, Jian‐Cai Chen, Xin Ge, Xiang‐Lin Chen, Xue‐Jiao Wang, Zhi‐Gang Yao, You‐Liang Tong, Bo Xie, Bai‐Hai Guo, Zhao‐Sheng Sun, Zhi‐Hua Tian, Ping Qiu, Xin‐Ru Lin, Qiu He, Shu‐Na Huang, Ke Ma, Fang‐Yu Wang, Huang‐Cheng Shang‐Guan, Wen‐Hua Fang, Deng‐Liang Wang, Ying Fu, Yuan‐Xiang Lin, De‐Zhi Kang

**Affiliations:** ^1^ Department of Neurosurgery Neurosurgery Research Institute Fujian Provincial Institutes of Brain Disorders and Brain Sciences National Regional Medical Center, First Affiliated Hospital and Binhai Campus, Fujian Medical University Fuzhou Fujian China; ^2^ Clinical Research and Translation Center National Regional Medical Center, First Affiliated Hospital and Binhai Campus, Fujian Medical University Fuzhou Fujian China; ^3^ Department of Neurosurgery Anxi County Hospital Quanzhou Fujian China; ^4^ Department of Critical Care Medicine Wuxi Ninth People's Hospital, Soochow University Wuxi Jiangsu China; ^5^ Department of Cerebrovascular Disease Qingyuan People's Hospital, The Sixth Affiliated Hospital of Guangzhou Medical University Qingyuan Guangdong China; ^6^ Department of Neurosurgery The Third People's Hospital of Datong Datong Shanxi China; ^7^ Department of Neurosurgery Shijiazhuang Third Hospital Shijiazhuang Hebei China; ^8^ Department of Neurosurgery Wuping County Hospital Longyan Fujian China; ^9^ Department of Neurosurgery Shishi General Hospital Quanzhou Fujian China; ^10^ Department of Neurology Jiuquan City People's Hospital Jiuquan Gansu China; ^11^ Department of Neurosurgery Harrison International Peace Hospital Hengshui Hebei China; ^12^ Department of Neurosurgery Jincheng People's Hospital Jincheng Shanxi China; ^13^ Department of Neurosurgery Longyan First Hospital Longyan Fujian China; ^14^ Department of Neurology Institute of Neurology National Regional Medical Center, First Affiliated Hospital and Binhai Campus, Fujian Medical University Fuzhou Fujian China

**Keywords:** intracerebral hemorrhage, long‐term, motor recovery, temporal profile

## Abstract

**Objective:**

Limited data is available to describe the temporal profile of long‐term recovery over 1 year after the stroke in patients with spontaneous intracerebral hemorrhage (ICH).

**Methods:**

A registered multicentral cohort was conducted to consecutively include non‐herniated supratentorial ICH patients from November 2013 to January 2023. Eligible patients received follow‐ups at the time of 3 months, 6 months, 1 year, and each year after the enrollments until death or the study termination. The outcome of motor recovery was assessed with the dichotomy of independent standing ability. Analyses were performed to investigate the associated factors, recovery rates, and temporal profile.

**Results:**

Of 1624 eligible responses, 105 (6.5%) regained motor recovery beyond 1 year after the stroke. The motor recovery course decreased with time and continued until 44 months, with 1‐year and long‐term cumulative recovery rates of 71.3% (95% CI: 69.0%–73.5%) and 80.2% (95% CI: 78.0%–82.5%), respectively. Moreover, the onset age, ICH location, larger ICH, and peripheral hematomal edema (PHE), intraventricular extension, GCS score, and admission hospital tier were independent factors on the motor outcome (all *p* < 0.05). However, the older age (*a*HR = 0.97/year, 95% CI: 0.95–0.98, *p* < 0.001) was identified as the only hazard factor for future recovery in patients who were incapable of recovery within 1 year.

**Interpretation:**

The poststroke recovery was ongoing beyond 1 year until about 3 years after the onset, and those with delayed motor recovery accounted for about 10% of ultimately recovered patients.

## 1 Introduction

Spontaneous intracerebral hemorrhage (ICH) accounts for a high prevalence of about 3.4 million per year and high disease burden, which becomes a major health problem worldwide [1, 2, 3]. Unfortunately, no significant progress has been available for neurological improvements in ICH patients in the past decades. Besides the irreversible pathophysiological changes from ICH, the treatment dilemma also attribute to the limited observation on diverse prognosis in ICH patients. As for outcome assessment in researches, it is worth mentioning about follow‐up time frames were mostly designed within 1 year after onset in current popular ICH cohorts and trials [4, 5, 6, 7, 8, 9, 10, 11, 12, 13, 14]. Whereas, this short timeframe of outcome assessment disregarded the ongoing motor recovery and might contribute to the misjudgment of ultimate recovery. Therefore, a better understanding of recovery course of motor function after ICH onset and associated factors for delayed recovery would be helpful, to identify patients with possible recovery beyond 1 year, and thereby to tailor more reasonable observational time frame in future clinical practice and researches.

The motor recovery after ICH could be varied depending on the longitudinal follow‐up time and severity of the original ICH. An ideal approach is to regularly assess the functional outcomes in the event of recovery or death, and get estimates for motor recovery within different time periods after ICH onset. However, limited data is available describing the temporal profile of motor recovery beyond 1 year. Therefore, the authors conducted annual follow‐ups on supratentorial ICH patients from a multicentral cohort. This work aims to reveal the full longitudinal picture of motor improvement in the entire and subgroup populations and identify associated characteristics for delayed motor recovery. Hopefully, our findings will provide a reference for the identification of possible recovery patients and the optimal timing of prognostic assessment.

## 2 Methods

### 2.1 Study Design and Population

The data for this analysis was obtained from a registered, nationwide, multicentral cohort study (ClinicalTrials.gov identifier: NCT03862729). Patients were enrolled from 23 sites, including 19 referral hospitals and four non‐referral hospitals across China. Eligible supratentorial spontaneous ICH patients were aged ≥ 18 years, diagnosed through skull computed tomography (CT) or CT angiography scans within 48 h of symptom onset or last‐seen‐well (LSW) time, and received the appropriate treatment according to the latest guidelines [15, 16, 17, 18, 19]. Patients with cerebral herniation at admission, infratentorial ICH, secondary ICH (resulted from underlying lesion, for example, vascular malformation, aneurysm, and tumor), premorbid modified Rankin Scale (mRS) score > 2, severe coagulopathy (PT‐INR incapable of reversing to ≤ 1.5), and pregnancy/lactation were excluded. Enrolled patients routinely received telephone follow‐ups for current outcomes and post‐discharge treatment. Follow‐ups were performed at the time of 3 months, 6 months, 1 year, and each year after the enrollment until death or the study termination.

### 2.2 Clinical Data Collection

Clinical data and assessment for each patient were documented on electronic case report forms (eCRF) and collected in an electronic data capture system (EDCs). Demographic information (age and sex), clinical characteristics (onset/LSW time and details), and premorbid history (comorbidities, medications, smoking, and drinking) were reviewed and recorded. During the follow‐up period, the post‐discharge treatment was reviewed, and survival status and prognostic scores were dynamically assessed and collected by investigators.

The severity of the stroke was assessed with the Glasgow Coma Scale (GCS) at admission. The admission hospital tier was recorded as a referral/non‐referral hospital. The referral hospitals were defined as grade‐A tertiary hospitals with a higher tier, receiving critical referrals and also primary emergencies. The non‐referral hospitals were any other medical centers and usually admitted primary emergencies. Surgical treatment indicated any surgical approach for hematoma evacuation or reducing intracranial hypertension during the admission (e.g., conventional craniotomy hematoma evacuation, endoscopic or catheter hematoma evacuation, and external ventricular drainage). The post‐discharge rehabilitation included receiving guidance from therapists in outpatient departments or rehabilitation institutions.

### 2.3 Radiologic Assessment

The radiological files were documented in the format of the Digital Imaging and Communications in Medicine (DICOM) for assessment. The baseline CT was performed at admission, and analyzed radiological features included ICH and PHE volumes, deep/lobar ICH location, and coexisting intraventricular hemorrhage (IVH). Moreover, at least three radiologists or clinicians participated in the radiological evaluation. Besides the principal evaluator, two additional evaluators participated in routine supervision or final judgment of contentious cases.

The volumetric measurements of ICH and PHE were determined using the ABC/2 formula [20], and the PHE volume was indirectly calculated by V_ICH+PHE_ – V_ICH_ [21]. To further investigate the effect of ICH and PHE volumes on the outcomes, analyzed patients were trichotomously stratified according to each 20 mL and categorized with small (< 20 mL), medium (20–40 mL), and large (≥ 40 mL) volumes.

### 2.4 Outcomes

In this work, the dichotomy of independent standing ability was the principal outcome of assessing motor recovery. We defined the independent stand as standing up without assistance (assistive devices permitted) and adopted this milestone event for functional recovery, for the better recall of this event in the intervals between annual follow‐ups. Notably, the dichotomy of outcome assessment minimized the bias during measurement in this long‐term survey.

Additionally, follow‐up time spanned from ICH onset/LSW to the first time of independent standing (for recovered patients) or the latest follow‐up (June 15th, 2023) (for those who are incapable of standing). For patients unable to accurately recall the time, the recovery duration was the midpoint of the recovery from last standing disability to the first regaining motor recovery.

### 2.5 Statistical Analyses

Statistical analyses were conducted using SPSS Statistics software (version 25.0, SPSS Inc., Chicago, USA) and R language software (version 4.1.0, Institute for Statistics and Mathematics, Vienna, Austria). All tests were two‐tailed, and *p* < 0.05 was considered statistically significant. For categorical data, the 𝝌^2^ test (continuity correction and Fisher's exact test, if necessary) was adopted for statistical analyses, while count and proportions [*n* (%)] were employed for statistical descriptions. For continuous variables with normal distribution, the student *t*‐test was employed for statistical analyses, and mean and standard deviation ($\bar{\chi }$±SD) were utilized for statistical descriptions. For highly skewed continuous variables, the Mann–Whitney *U* test was adopted for statistical analyses, and the median with interquartile ranges (median, IQR) was utilized for statistical descriptions.

For the description of the temporal profile, the Kaplan–Meier (K‐M) survival estimate was performed to construct the cumulative probability curves and calculate the recovery time. And the life table was adopted for cumulative recovery rates. Subgroup analyses were further conducted to demonstrate data according to the ICH/PHE volumes and ICH locations (deep/lobar ICH). The log‐rank method and the Benjamini–Hochberg (BH) method (pairwise comparisons) were for the detection whether the motor outcome differed between/among subgroups.

The Cox proportional hazards regression model was used to explore the associated factors on the long‐term and 1‐year motor outcomes of all analyzed ICH patients during the recovery course, and the future recovery in patients who were incapable of recovery within 1 year. The results were presented as adjusted hazard ratios (*a*HRs) with 95% confidence intervals (CIs).

### 2.6 Ethics

The study was conducted in accordance with the Declaration of Helsinki and approved by ethics committees or institutional review boards at each site (MRCTA, ECFAH of FMU [2018]082). Informed consent was obtained from patients or their guardians who could not consent during enrollments, and verbal consent was obtained during the follow‐ups for the retrospective enrollments.

## 3 Results

### 3.1 Baseline Characteristics

**FIGURE 1:**
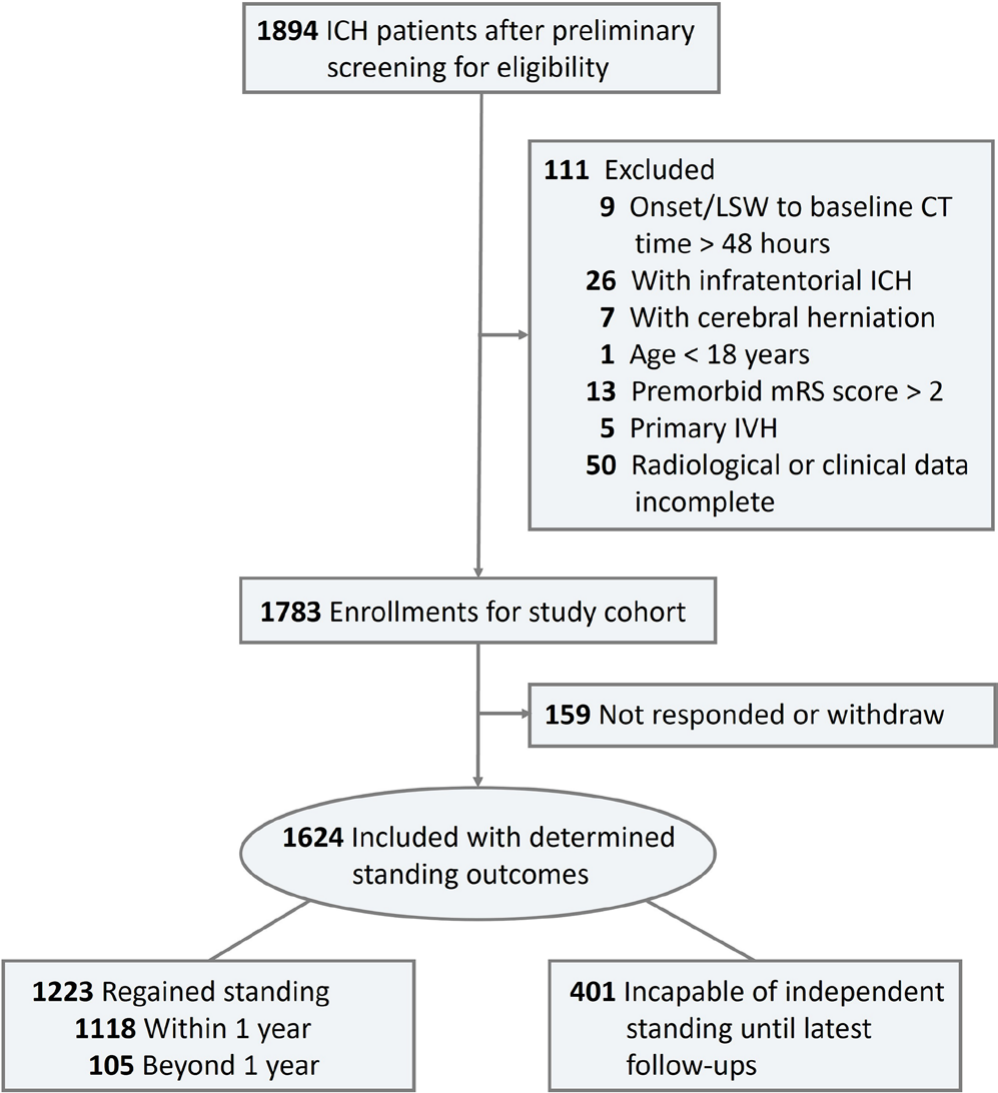
Flow diagram depicting study population. CT, computed tomography; IVH, intraventricular hemorrhage; mRS, modified Rankin scale.

A total of 1894 ICH patients were preliminary screened for eligibility spanning from November 2013 to January 2023. Out of 1783 ICH patients who met the criteria, 159 (8.9%) patients did not respond to follow‐ups, and 1624 eligible patients successfully received follow‐ups with determined motor outcomes for analyses (Figure 1). The median follow‐up time was 32.6 (95% CI: 29.3–35.9) months, and the maximum follow‐up was 91 months.

Among the entire analyzable population, the mean age was 60.7 ± 12.7 years, with 533 (32.8%) females (Table 1). A total of 1223 (75.3%) ICH patients regained motor recovery, whereas 401 (24.7%) remained unrecovered motor function until the latest follow‐up. The mean age of the recovered group was younger than the unrecovered group (58.7 ± 12.2 years vs. 66.8 ± 12.4 years, *p* < 0.001). The sex composition of the two groups was comparable (female: 33.8% vs. 29.9%, *p* = 0.16). The patients had a larger median ICH volume of 26.5 (12.0–47.6) mL in the unrecovered group than in the recovered group with 9.7 (4.6–19.4) mL (*p* < 0.001).

**TABLE 1:** Clinical characteristics and univariate Cox proportional hazards regression model for standing outcome.

Characteristics	Recovered group (*n* = 1223)	Unrecovered group (*n* = 401)	Univariate analysis	Univariate Cox regression	
			*p*	HR (95% CI)	*p*
Age, mean (SD), years	58.7 (12.2)	66.8 (12.4)	< 0.001	0.98 (0.98–0.99)	< 0.001
Onset/LSW to baseline CT time, median (IQR), hours	4.6 (2.2–11.7)	4.3 (2.2–8.7)	0.04	1.01 (1.00–1.01)	0.007
Admission to referral hospitals	945 (77.3)	330 (82.3)	0.04	0.78 (0.69–0.90)	< 0.001
Sex					
Male	810 (66.2)	281 (70.1)	0.16	Reference	—
Female	413 (33.8)	120 (29.9)		1.09 (0.97–1.22)	0.17
Smoking					
Never	964 (78.8)	325 (81.9)	0.54	Reference	—
Occasionally	22 (1.8)	7 (1.8)		0.92 (0.60–1.40)	0.69
Regular	197 (16.1)	56 (14.1)		1.12 (0.96–1.30)	0.16
Cessation	40 (3.3)	9 (2.3)		1.21 (0.88–1.65)	0.25
Drinking					
Never	970 (79.4)	332 (83.4)	0.24	Reference	—
Occasionally	89 (7.3)	27 (6.8)		1.10 (0.89–1.37)	0.38
Regular	152 (12.4)	35 (8.8)		1.17 (0.99–1.39)	0.07
Cessation	11 (0.9)	4 (1.0)		1.16 (0.64–2.10)	0.62
Diabetes	127 (10.4)	52 (13.0)	0.17	0.90 (0.75–1.08)	0.27
Hypertension	854 (70.1)	285 (72.3)	0.41	0.95 (0.84–1.08)	0.45
Premorbid mRS score					
0	1122 (91.7)	342 (85.5)	< 0.001	Reference	—
1	97 (7.9)	51 (12.8)		0.76 (0.62–0.93)	0.009
2	4 (0.3)	7 (1.8)		0.47 (0.18–1.26)	0.13
Premorbid medication					
Antiplatelet drugs	21 (1.7)	23 (5.8)	< 0.001	0.50 (0.32–0.76)	0.001
Anticoagulant drugs	8 (0.7)	4 (1.0)	0.50	0.73 (0.36–1.46)	0.38
GCS score, point	14 (12–15)	11 (8–13)	< 0.001	1.23 (1.20–1.26)	< 0.001
13–15	874 (71.5)	130 (32.4)	< 0.001	Reference	—
9–12	264 (21.6)	142 (35.4)		0.44 (0.39–0.51)	< 0.001
3–8	85 (7.0)	129 (32.2)		0.22 (0.17–0.27)	< 0.001
ICH location					
Lobar	183 (15.0)	38 (9.5)	< 0.001	Reference	—
Deep	1040 (85.0)	363 (90.5)		0.71 (0.61–0.83)	< 0.001
Intraventricular hemorrhage	333 (27.2)	213 (53.1)	< 0.001	0.53 (0.47–0.60)	< 0.001
Surgical treatment	234 (19.1)	177 (44.1)	< 0.001	0.45 (0.39–0.52)	< 0.001
ICH volume, median (IQR), mL	9.7 (4.6–19.4)	26.5 (12.0–47.6)	< 0.001	0.97 (0.97–0.98)	< 0.001
Small (< 20 mL)	922 (75.4)	159 (39.7)	< 0.001	Reference	—
Medium (20–40 mL)	191 (15.6)	113 (28.2)		0.48 (0.41–0.56)	< 0.001
Large (≥ 40 mL)	110 (9.0)	129 (32.2)		0.32 (0.26–0.39)	< 0.001
PHE volume, median (IQR), mL	5.6 (2.4–11.8)	13.4 (6.2–23.8)	< 0.001	0.97 (0.96–0.97)	< 0.001
Small (< 20 mL)	1077 (88.1)	261 (65.1)	< 0.001	Reference	—
Medium (20–40 mL)	116 (9.5)	100 (24.9)		0.46 (0.38–0.55)	< 0.001
Large (≥ 40 mL)	30 (2.5)	40 (10.0)		0.33 (0.23–0.48)	< 0.001
Post‐discharge rehabilitation					
No received	565 (46.2)	81 (20.3)	< 0.001	Reference	—
Received	464 (37.9)	83 (20.8)		0.74 (0.65–0.83)	< 0.001
Unknown	194 (15.9)	235 (58.9)		0.34 (0.29–0.40)	< 0.001

Abbreviations: CT, computed tomography; GCS, Glasgow Coma Scale; HR, hazard ratio; ICH, intracerebral hemorrhage; IQR, interquartile range; LSW, last seen well; mRS, modified Rankin scale; PHE, perihematomal edema; SD, standard deviation.

### 3.2 Temporal Profile of Poststroke Motor Recovery

**FIGURE 2:**
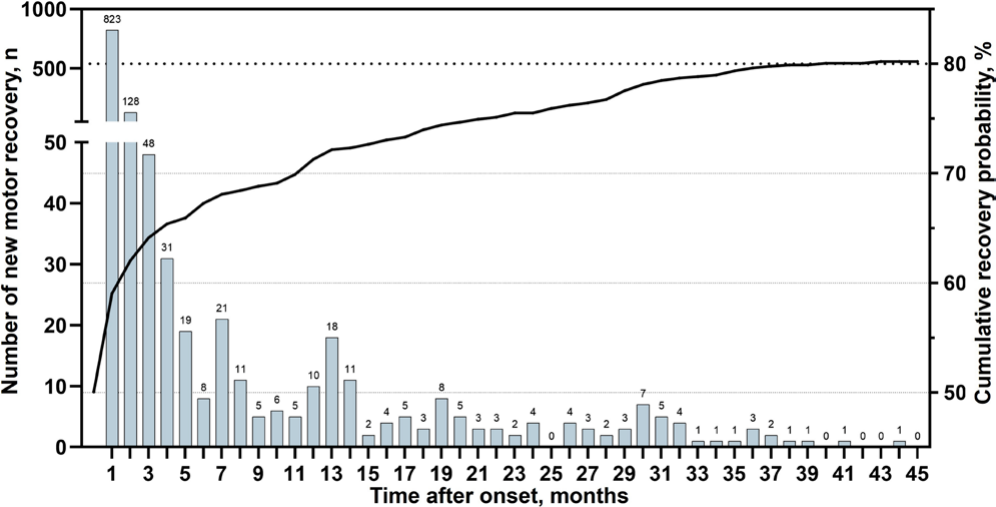
Incident of post‐hemorrhagic motor recovery and Kaplan–Meier curves.

Most patients regained motor recovery in the first month, and the median recovery time was 1.0 (95% CI: 0.8–1.1) months after the stroke. Out of 1624 ICH patients with the determined outcome, 1118 (68.8%) regained motor recovery within 1 year after the stroke, and the motor recovery events decreased with time and continued until 44 months (Figure 2). And 105 (6.5%) patients regained standing up beyond 1 year after the stroke, accounting for almost 10% of ultimate motor recovery. As calculated by the life table, the cumulative recovery rates of ICH patients after onset were as follows: 64.1% (95% CI: 61.8%–66.5%) within 3 months, 67.3% (95% CI: 65.0%–69.6%) within 6 months, 71.3% (95% CI: 69.0%–73.5%) within 1 year, 75.5% (95% CI: 73.3%–77.7%) within 2 years, 79.6% (95% CI: 77.4–81.8) within 3 years, 80.2% (95% CI: 78.0–82.5) with ultimate motor recovery (Figure 2 and Table S1).

**FIGURE 3:**
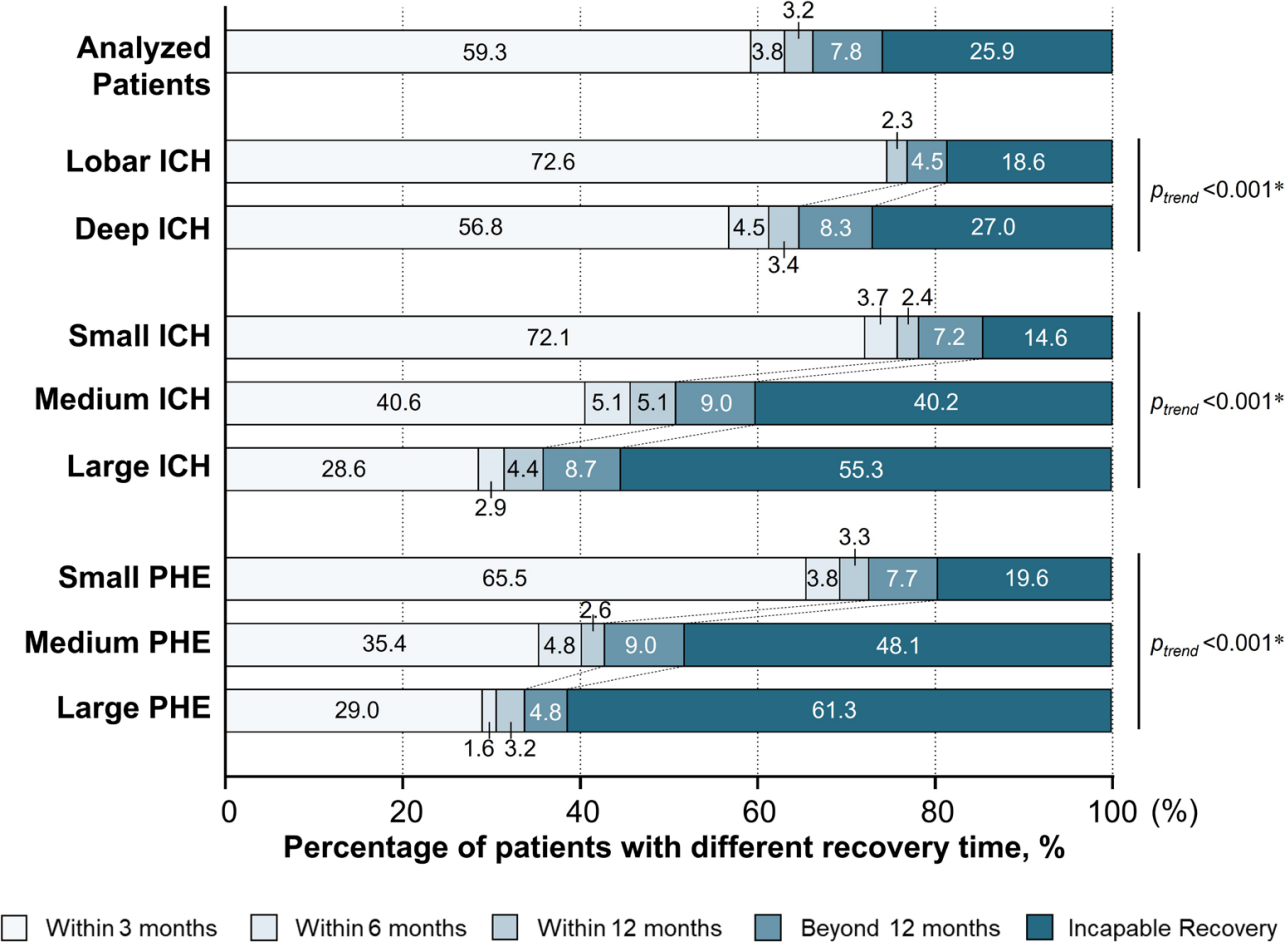
Percentages of patients with different motor recovery times. ICH, intracerebral hemorrhage; PHE, perihematomal edema. *The *p* value was calculated by the chi‐square test for trend.

**FIGURE 4:**
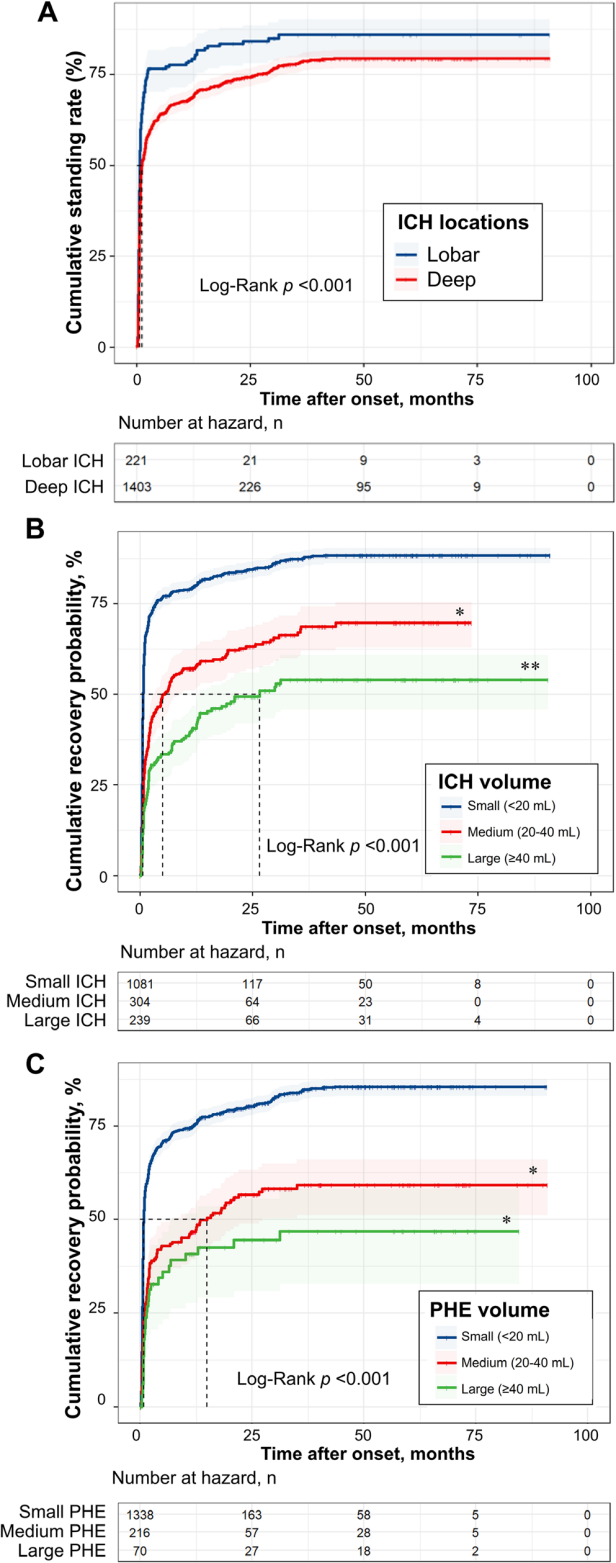
Kaplan–Meier curves for motor recovery in subgroups by (A) ICH location, (B) ICH volume categories, and (C) PHE volume categories. ICH, intracerebral hemorrhage; PHE, perihematomal edema. *Adjusted *p* value < 0.05 compared with small ICH/PHE group (< 20 mL), by BH method. **Adjusted *p* value < 0.05 compared with both small and median ICH/PHE groups (< 20 mL and 20–40 mL), by BH method.

Subgroup analyses were further performed to demonstrate temporal profiles of motor recovery in patients stratified by ICH locations, ICH and PHE volume categories. The percentages of patients with different recovery times and motor recovery probabilities in each group were illustrated in Figures 3 and 4. Compared with lobar location, the median standing time was longer and the cumulative standing rate was lower in patients with deep ICH (Log‐rank *p* < 0.001; Figure 4A, Tables S1–S4). As the higher ICH or PHE volume category referred, there were trends of delayed motor recovery and longer median recovery time (Log‐rank *p* < 0.001; Figure 4B,C, Tables S1–S4). The data for interacted groups was available in Table S1 and Figures S1 and S2.

### 3.3 Associated Factors for Long‐Term Motor Recovery

Adjusted with the inconsistent time of outcome assessment, the older age, lower GCS score, larger ICH/PHE volume, and premorbid antiplatelet medication were identified as hazard factors for ultimate motor disability by Cox regression (Table 1). Besides, surgical treatment was a significant interventional factor for deteriorated motor recovery (HR = 0.45, 95% CI: 0.39–0.52, *p* < 0.001) but did not remain an independent contributor in the further multivariate model (*a*HR = 0.91, 95% CI: 0.77–1.09, *p* = 0.31). The onset age, ICH location, ICH/PHE volume, coexisting IVH, GCS score, and admission hospital tier were identified as independent factors for long‐term motor recovery (Table 2).

**TABLE 2:** Multivariable Cox proportional hazards regression model for standing outcome.

Characteristics	*a*HR (95% CI)	Statistics	*p*
Age, per years	0.98 (0.97–0.98)	81.26	< 0.001
Onset/LSW to baseline CT time, per hour	1.00 (1.00–1.01)	1.23	0.27
Admission to referral hospitals	0.82 (0.72–0.94)	7.89	0.005
Premorbid mRS score			
0	Reference	—	—
1	0.84 (0.68–1.04)	2.59	0.11
2	0.68 (0.25–1.83)	0.58	0.45
Antiplatelet drugs	0.66 (0.43–1.03)	3.39	0.07
GCS score			
13–15	Reference	—	—
9–12	0.57 (0.49–0.66)	58.87	< 0.001
3–8	0.43 (0.34–0.55)	47.24	< 0.001
Deep ICH location	0.55 (0.47–0.65)	48.53	< 0.001
Intraventricular hemorrhage	0.69 (0.61–0.79)	29.64	< 0.001
Surgical treatment	0.91 (0.77–1.09)	1.01	0.31
ICH volume, per mL	0.98 (0.98–0.99)	44.62	< 0.001
PHE volume, per mL	0.99 (0.98–1.00)	5.77	0.016
Post‐discharge rehabilitation			
No received	Reference	—	—
Received	0.84 (0.74–0.95)	7.58	0.006
Unknown	0.52 (0.44–0.62)	56.46	< 0.001

Abbreviations: *a*HR, adjusted hazard ratio; CI, confidence interval; CT, computed tomography; GCS, Glasgow coma scale; ICH, intracerebral hemorrhage; LSW, last seen well; mRS, modified Rankin scale; PHE, perihematomal edema.

Associated factors on 1‐year motor outcome were identified and demonstrated in Tables S5 and S6, which were comparable to the above characteristics for the ultimate motor recovery.

### 3.4 Delayed Recovery Hazard After 1 Year

Further, analyses were performed to identify possible future recovery in patients who were incapable of recovery within 1 year after the stroke. The characteristics were compared between patients with delayed recovery beyond 1 year and those without ever recovery. It was observed that older age, lower GCS score, larger ICH and PHE volume, and coexisting IVH were identified as hazard factors for ultimate motor outcome in these patients (Table 3). Further multivariate Cox regression shows that the hazard of motor recovery decreased as the age increased (*a*HR = 0.97/year, 95% CI: 0.95–0.98, *p* < 0.001; Table 4), which remained as the only independent factor for future recovery in patients who were incapable of recovery within 1 year. The undetermined status of post‐discharge rehabilitation was excluded from the analysis.

**TABLE 3:** Clinical characteristics and univariate Cox proportional hazards regression model in patients without 1‐year recovery.

Characteristics	Recovered group (*n* = 105)	Unrecovered group (*n* = 401)	Univariate analysis	Univariate Cox regression	
			*p*	HR (95% CI)	*p*
Age, mean (SD), years	58.4 (10.9)	66.79 (12.37)	< 0.001	0.95 (0.94–0.97)	< 0.001
Onset/LSW to baseline CT time, median (IQR), hours	5.8 (2.1–12.0)	4.3 (2.2–8.7)	0.07	1.01 (0.99–1.03)	0.48
Admission to referral hospitals	99 (94.3)	330 (82.3)	0.004	1.90 (0.83–4.36)	0.13
Sex					
Male	72 (68.6)	281 (70.1)	0.81	Reference	—
Female	33 (31.4)	120 (29.9)		0.99 (0.66–1.50)	0.98
Smoking					
Never	86 (81.9)	325 (81.9)	0.74	Reference	—
Occasionally	1 (1)	7 (1.8)		0.53 (0.07–3.81)	0.53
Regular	17 (16.2)	56 (14.1)		1.09 (0.65–1.83)	0.75
Cessation	1 (1)	9 (2.3)		0.38 (0.05–2.71)	0.33
Drinking					
Never	85 (81)	332 (83.4)	0.79	Reference	—
Occasionally	6 (5.7)	27 (6.8)		0.93 (0.41–2.12)	0.86
Regular	13 (12.4)	35 (8.8)		1.48 (0.82–2.65)	0.19
Cessation	1 (1)	4 (1)		1.46 (0.20–10.5)	0.71
Diabetes	10 (9.5)	52 (13)	0.41	0.83 (0.43–1.59)	0.57
Hypertension	69 (65.7)	285 (72.3)	0.23	0.68 (0.46–1.02)	0.06
Premorbid mRS score					
0	96 (91.4)	342 (85.5)	0.17	Reference	
1	9 (8.6)	51 (12.8)		0.68 (0.34–1.34)	0.26
2	0	7 (1.8)		NA^a^	NA^a^
Premorbid medication					
Antiplatelet drugs	2 (1.9)	23 (5.8)	0.13	0.56 (0.14–2.28)	0.42
Anticoagulant drugs	1 (1)	4 (1)	> 0.99	0.63 (0.09–4.54)	0.65
GCS score, point	13 (10–14)	11 (8–13)	< 0.001	1.13 (1.06–1.20)	< 0.001
13–15	53 (50.5)	130 (32.4)	< 0.001	Reference	—
9–12	37 (35.2)	142 (35.4)		0.72 (0.47–1.09)	0.12
3–8	15 (14.3)	129 (32.2)		0.34 (0.19–0.61)	< 0.001
ICH location					
Lobar	10 (9.5)	38 (9.5)	> 0.99	Reference	—
Deep	95 (90.5)	363 (90.5)		0.93 (0.48–1.78)	0.82
Intraventricular hemorrhage	33 (31.4)	213 (53.1)	< 0.001	0.45 (0.30–0.67)	< 0.001
Surgical treatment	34 (32.4)	177 (44.1)	0.03	0.81 (0.54–1.21)	0.30
ICH volume, median (IQR), mL	13.5 (8.0–31.0)	26.5 (12.0–47.6)	< 0.001	0.98 (0.97–0.99)	0.001
Small (< 20 mL)	65 (61.9)	159 (39.7)	< 0.001	Reference	—
Medium (20–40 mL)	21 (20)	113 (28.2)		0.59 (0.36–0.97)	0.04
Large (≥ 40 mL)	19 (18.1)	129 (32.2)		0.54 (0.32–0.90)	0.02
PHE volume, median (IQR), mL	7.7 (3.4–17.4)	13.4 (6.2–23.8)	< 0.001	0.97 (0.96–0.99)	0.001
Small (< 20 mL)	84 (80)	261 (65.1)	0.008	Reference	—
Medium (20–40 mL)	18 (17.1)	100 (24.9)		0.62 (0.37–1.03)	0.07
Large (≥ 40 mL)	3 (2.9)	40 (10)		0.22 (0.07–0.71)	0.01
Post‐discharge rehabilitation					
No received	46 (43.8)	81 (20.3)	< 0.001	Reference	—
Received	56 (53.3)	83 (20.8)		1.37 (0.93–2.03)	0.11
Unknown	3 (2.9)	235 (58.9)		0.02 (0.01–0.08)	< 0.001

Abbreviations: CT, computed tomography; GCS, Glasgow Coma Scale; HR, hazard ratio; ICH, intracerebral hemorrhage; IQR, interquartile range; LSW, last seen well; mRS, modified Rankin scale; PHE, perihematomal edema; SD, standard deviation.

^a^ Hazard ratio was incapable due to less sample size in the corresponding group.

**TABLE 4:** Multivariate Cox proportional hazards regression model in patients without 1‐year recovery.

Characteristics	*a*HR (95% CI)	Statistics	*p*
Age, per year	0.97 (0.95, 0.98)	14.83	< 0.001
GCS score			
13–15	Reference	—	—
9–12	0.82 (0.53, 1.27)	0.80	0.37
3–8	0.70 (0.36, 1.33)	1.22	0.27
Intraventricular hemorrhage	0.69 (0.45, 1.07)	2.68	0.10
ICH volume, per mL	0.99 (0.98, 1.00)	2.79	0.10
PHE volume, per mL	1.00 (0.98, 1.02)	0.12	0.73
Post‐discharge rehabilitation			
No received	Reference	—	—
Received	1.09 (0.72, 1.65)	0.17	0.68
Unknown	0.03 (0.01, 0.10)	33.89	< 0.001

Abbreviations: *a*HR, adjusted hazard ratio; GCS, Glasgow Coma Scale; ICH, intracerebral hemorrhage; PHE, perihematomal edema.

## 4 Discussion

This work revealed that the long‐term motor recovery continued beyond 1 year within about 3 years after the stroke, with an overall independent recovery rate of about 75% and a 1‐year rate of about 69% in supratentorial ICH patients. Moreover, the onset age, ICH location, larger ICH/PHE, intraventricular extension, GCS score, and admission hospital tier were independent factors on the motor outcome. As for patients who were incapable of recovery within 1 year, older age was identified as the only hazard factor for future recovery.

Over the past decades, several randomized controlled trials have been conducted for ICH treatment, while there was no substantial neurological improvement from scoped medical or surgical intervention. However, most were designed with follow‐up durations between 3 months and 1 year, and the long‐term follow‐ups (> 1 year) were rarely implemented in current large cohorts (Table S7) [4, 5, 6, 7, 8, 9, 10, 11, 12, 13, 14]. It was still unclear about the consistency between prognostic assessment within 1 year (particularly within 3 months) and ultimate motor outcomes [22, 23, 24], and limited studies with long‐term follow‐ups were available to describe the temporal profile of poststroke motor recovery [25, 26, 27, 28].

Differently, this work performed a long‐term follow‐up with a median time of over 32 months and a maximum time of up to 91 months and reported the temporal profile of this long‐term motor recovery course instead of simply reporting lateral sectioned outcomes within a specific time window. The description of a continued motor recovery course might benefit in tailoring a more reasonable assessment of timing as stable neurological outcomes. Lee et al. reported that independent ambulation could continue to improve within only 6 months [22], although it remained unclear of the possible time window of motor recovery beyond conventional observation within 1 year in current studies. In this work, the authors observed that some ICH patients continued rehabilitating and regained motor recovery beyond 1 year after the stroke, and these delayed recovered patients accounted for about 10% of patients with ultimate recovery. While motor recovery nearly stagnated beyond 3 years after the stroke and remained the ultimate standing dependency, accounting for about 25% of the analyzed population. This result indicated that motor recovery may continue beyond the conventional observational time frame, and rehabilitation should be insistent in delayed recovered patients for possible improvement. Moreover, the present assessment of prognosis within 1 year might confound the stable outcomes for these delayed recovered patients, and conventional timing of assessments might result in the misjudgment of true recovery and false negative samples in practice. Thus, the authors assumed that the ultimate and stable neurological outcomes should be observed for 3 years rather than 1 year or even a few months, in future studies.

In this observational cohort, the motor recovery rate within 3–6 months (about 61%–65% of the follow‐up ICH patients) was comparable to the percentages reported in the *ATACH‐II* and *CLEAR‐III* trials (principle neurological outcome: mRS ≤ 3) by rough comparisons [8, 13]. Nonetheless, it must be acknowledged that China is a developing country with establishing health systems and unequally distributed medical resources, and people's health concepts are deficient (especially in the older population). Therefore, the recovery rates may be overestimated in this work, compared to the prognostic reports in Western countries. This result could be explained by the inconsistent definition of favorable outcomes (motor recovery vs. mRS ≤ 3) and the different enrolling eligibility (infratentorial or critical herniated ICH exclusion vs. comprehensive enrollment).

It has been admitted that standing recovery is a seldom used tool for neurological recovery [29], the status of regaining standing ability is comparable to a neurological function between mRS 3 and 4. Although it is no superior in precise grading of neurological outcomes compared to mRS score, the assessment of standing ability was more practical and intuitive in our long‐term survey to demonstrate the temporal profile of motor recovery. Furthermore, the milestone event of standing can reduce bed‐rest complications, enhance patients' confidence in insistent rehabilitation, and thus possesses certain values.

Previous reports indicated that the location and ICH/PHE volume play an important role in neurological outcomes [30, 31, 32, 33, 34]. The authors explored different trends in the temporal profile of motor recovery in subgroup analyses by these indicators. In agreement with prior research, patients trended to have delayed motor recovery or even debilitated motor outcomes in subgroups with deep locations or larger ICH/PHE volumes. However, there existed patients who continued to regain motor recovery beyond 1 year after the stroke, no matter in each subgroup. In analyses of recovery hazard after 1 year, the onset age was identified as the only independent factor for future recovery. Thus, the long‐term motor recovery should be noticed and the persistent rehabilitation would be significant in all ICH patients, particularly those who were incapable of recovery within 1 year. These highlighted factors should be considered in the future clinical trial design for motor recovery, especially in different stages after stroke.

This work interpreted the temporal profile of post‐hemorrhagic motor recovery and provided cortical data for the identification of possible recovery patients and the optimal timing of prognostic assessment. However, there are limitations that deserve comment in this work. Notably, the profile is not comprehensive for all motor‐impaired patients due to the exclusion of brainstem ICH or cerebral herniation. These critical patients were likely to have an unfavorable outcome of death or lifetime chair dependency, and the exclusion might cause the overestimation of early motor recovery. Hence, it requires a longer survey in larger samples for comprehensive description in future studies. On the other hand, there were biases affecting the reliability of our findings. Although we introduced the dichotomized assessment of the milestone events to minimize the bias. Despite this, the prospective design and those measures could not eliminate the recall bias in the gap between each follow‐up in this long‐term survey. Thus, the authors recommend using the findings and conclusions presented in this work with caution.

## 5 Conclusion

The motor recovery decreased with time and continued for about 3 years after the stroke, and the patients with delayed motor recovery accounted for about 10% of those with ultimate recovery. Absolutely, the long‐term motor recovery over 1 year should be noticed in future clinical practice and study design, and the persistent rehabilitation would be significant, particularly for those who were incapable of recovery within 1 year.

## Author Contributions

Wen‐Hua Fang, Deng‐Liang Wang, Ying Fu, Yuan‐Xiang Lin, and De‐Zhi Kang were involved in conceptualization. Fu‐Xin Lin, Ling‐Yun Zhuo, and Ying Fu were involved in methodology. Ke Ma was involved in software. Xin‐Ru Lin, Shu‐Na Huang, and Ke Ma were involved in data curation and supervision. Fu‐Xin Lin, Yan Zheng, Jian‐Cai Chen, Xin Ge, Xiang‐Lin Chen, Xue‐Jiao Wang, Zhi‐Gang Yao, You‐Liang Tong, Bo Xie, Bai‐Hai Guo, Zhao‐Sheng Sun, Zhi‐Hua Tian, Ping Qiu, Qiu He, Fang‐Yu Wang, and Huang‐Cheng Shang‐Guan were involved in investigation. Ling‐Yun Zhuo was involved in formal analysis. De‐Zhi Kang was involved in funding acquisition. Yan Zheng and Ling‐Yun Zhuo were involved in visualization. Fu‐Xin Lin and Qiu He were involved in project administration. Yuan‐Xiang Lin and De‐Zhi Kang were involved in resources. Fu‐Xin Lin, Yan Zheng, and Ling‐Yun Zhuo were involved in writing – original draft. Yan Zheng, Ling‐Yun Zhuo, Ying Fu, Yuan‐Xiang Lin, and De‐Zhi Kang were involved in writing – review and editing.

## Conflicts of Interest

The authors declare no conflicts of interest.

## Supporting information


**Table S1.** The Kaplan–Meier estimation for recovery times and cumulative recovery rates.
**Figure S1.** Subgroup analyses of post‐hemorrhagic motor recovery by ICH volume.
**Figure S2.** Subgroup analyses of post‐hemorrhagic motor recovery by PHE volume.
**Table S2.** Patient count of first motor recovery within poststroke time by ICH volume.
**Table S3.** Patient count of first motor recovery within poststroke time by PHE volume.
**Table S4.** Patient count of first motor recovery within poststroke time by ICH locations.
**Table S5.** Clinical characteristics and Cox proportional hazards regression models by 1‐year motor outcomes.
**Table S6.** Multivariable Cox proportional hazards regression model by 1‐year motor outcomes.
**Table S7.** Outcome assessments in popular studies.

## Data Availability

Deidentified case data are available from the authors upon reasonable request, after request in writing to the corresponding authors and after approval by the study committee and principal investigators from each site.
