# Supplementary material for: Long‐Term Temporal Profile of Motor Recovery After Intracerebral Hemorrhage
Source: Ann Clin Transl Neurol. 2025 Aug 5;12(11):2235–45. doi: 10.1002/acn3.70124 (PMC12623826; doi:10.1002/acn3.70124)
Supplement: Supplementary file 1 — Table S1. The Kaplan–Meier estimation for recovery times and cumulative recovery rates. Figure S1. Subgroup analyses of post‐hemorrhagic motor recovery by ICH volume. Figure S2. Subgroup analyses of post‐hemorrhagic motor recovery by PHE volume. Table S2. Patient count of first motor recovery within poststroke time by ICH volume. Table S3. Patient count of first motor recovery within poststroke time by PHE volume. Table S4. Patient count of first motor recovery within poststroke time by ICH locations. Table S5. Clinical characteristics and Cox proportional hazards regression models by 1‐year motor outcomes. Table S6. Multivariable Cox proportional hazards regression model by 1‐year motor outcomes. Table S7. Outcome assessments in popular studies. [file ACN3-12-2235-s001.docx]

# Supplemental Material

This supplemental material contains the following items:

[**Table S1:** The Kaplan-Meier estimation for recovery times and cumulative recovery rates](#_Table_S1._Demographic,)

[**Figure S1:** Subgroup analyses of post-hemorrhagic motor recovery by ICH volume](#_Figure_S1:_Subgroup)

[**Figure S2:** Subgroup analyses of post-hemorrhagic motor recovery by PHE volume](#_Figure_S2:_Subgroup)

[**Table S2:** Patient count of first motor recovery within post-stroke time by ICH volume](#_Table_S4._Patient)

[**Table S3:** Patient count of first motor recovery within post-stroke time by PHE volume](#_Table_S3:_Patient)

[**Table S4:** Patient count of first motor recovery within post-stroke time by ICH locations](#_Table_S4:_Patient)

[**Table S5:** Clinical characteristics and Cox proportional hazards regression models by 1-year motor outcomes](#_Table_S5:_Clinical)

[**Table S6:** Multivariable Cox proportional hazards regression model by 1-year motor outcomes](#_Table_S6:_Multivariable)

[**Table S7:** Outcome assessments in popular studies](#_Table_S7:_Outcome)

# Table S1: The Kaplan-Meier estimation for recovery times and cumulative recovery rates

| **Recovery times and rates** | **Entire analyzed population** | **ICH volume** | | | **PHE volume** | | |
| --- | --- | --- | --- | --- | --- | --- | --- |
|  |  | **Small**  **(<20 mL)** | **Medium**  **(20–40 mL)** | **Large**  **(≥40 mL)** | **Small**  **(<20 mL)** | **Medium**  **(20–40 mL)** | **Large**  **(≥40 mL)** |
| ***Entire analyzed population*** |  |  |  |  |  |  |  |
| Median follow-up time, months | 32.6 (29.3–35.9) | 41.3 (32.6–50.0) | 29.7 (23.8–35.5) | 27.7 (22.2–33.2) | 32.3 (28.8–35.7) | 31.3 (23.6–39.0) | 48.3 (28.4–68.3) |
| Mean recovery time, months | 21.3 (19.4–23.2) | 13.4 (11.6–15.3) | 26.7 (22.7–30.6) | 45.4 (39.4–51.4) | 16.7 (14.8–18.5) | 40.4 (34.3–46.5) | 47.1 (37.2–57.1) |
| Median recovery time, months | 1.0 (0.8–1.1) | 0.7 (0.6–0.7) | 5.1 (2.7–7.5) | NA***** | 0.8 (0.7–0.8) | 14.9 (7.7–22.1) | NA***** |
| 3-month recovery rate, % | 64.1 (61.8–66.5) | 74.1 (71.6–76.7) | 44.1 (38.4–49.8) | 30.6 (24.7–36.5) | 67.4 (64.9–69.9) | 38.9 (32.4–45.4) | 32.8 (21.6–44.0) |
| 6-month recovery rate, % | 67.3 (65.0–69.6) | 77.2 (74.5–79.8) | 50.8 (45.1–56.5) | 33.5 (27.4–39.6) | 71.2 (68.8–73.6) | 43.0 (36.3–49.7) | 36.0 (24.4–47.6) |
| 1-year recovery rate, % | 71.3 (69.0–73.5) | 79.8 (77.5–82.2) | 57.1 (51.4–62.8) | 40.6 (34.1–47.1) | 75.2 (72.8–77.6) | 46.7 (39.8–53.6) | 40.8 (28.8–52.8) |
| 2-year recovery rate, % | 75.5 (73.3–77.7) | 84.5 (85.3–86.7) | 63.2 (57.4–68.9) | 49.5 (42.4–56.6) | 80.2 (78.0–82.4) | 56.6 (49.3–63.9) | 44.6 (32.4–56.8) |
| 3-year recovery rate, % | 79.6 (77.4–81.8) | 87.5 (85.3–89.7) | 68.7 (62.6–74.8) | 54.0 (46.6–61.5) | 84.4 (82.2–86.6) | 59.3 (52.0–66.6) | 46.9 (34.4–59.4) |
| Long-term recovery rate, % | 80.2 (78.0–82.5) | 88.4 (86.2–90.6) | 69.8 (63.5–76.1) | 54.0 (46.6–61.5) | 85.5 (83.3–87.7) | 59.3 (52.0–66.6) | 46.9 (34.4–59.4) |
| ***Labor ICH location*** |  |  |  |  |  |  |  |
| Mean recovery time, months | 14.9 (10.5–19.3) | 11.2 (5.8–16.6) | 10.4 (3.7–17.1) | 21.6 (11.9–31.4) | 10.3 (5.6–15.0) | 20.8 (11.3–30.2) | 23.7 (9.1–38.3) |
| Median recovery time, months | 0.7 (0.6–0.7) | 0.6 (0.5–0.6) | 0.7 (0.6–0.8) | 1.4 (0.5–2.4) | 0.6 (0.5–0.6) | 1.2 (0.4–2.1) | 1.8 (0.9–2.6) |
| 3-month recovery rate, % | 76.6 (71.0–82.3) | 83.8 (76.7–90.9) | 81.8 (71.6–92.0) | 59.1 (46.6–71.6) | 84.3 (78.2–90.4) | 64.6 (51.7–77.5) | 60.0 (40.8–79.2) |
| 6-month recovery rate, % | 76.6 (71.0–82.3) | 83.8 (76.7–90.9) | 81.8 (71.6–92.0) | 59.1 (46.6–71.6) | 84.3 (78.2–90.4) | 64.6 (51.7–77.5) | 60.0 (40.8–79.2) |
| 1-year recovery rate, % | 78.7 (73.2–84.2) | 83.8 (76.7–90.9) | 81.8 (71.6–92.0) | 66.9 (54.6–79.2) | 84.3 (78.2–90.4) | 70.9 (58.6–83.2) | 64.4 (45.4–83.4) |
| 2-year recovery rate, % | 84.1 (79.0–89.3) | 88.1 (81.8–94.4) | 88.6 (78.6–98.6) | 73.6 (61.6–85.6) | 89.2 (83.7–94.7) | 78.0 (66.2–89.8) | 68.9 (50.5–87.3) |
| 3-year recovery rate, % | 85.9 (80.8–91.0) | 89.3 (83.2–95.4) | 88.6 (78.6–98.6) | 78.9 (65.6–92.2) | 90.5 (85.0–96.0) | 78.0 (66.2–89.8) | 76.7 (57.5–95.9) |
| Long-term recovery rate, % | 85.9 (80.8–91.0) | 89.3 (83.2–95.4) | 88.6 (78.6–98.6) | 78.9 (65.6–92.2) | 90.5 (85.0–96.0) | 78.0 (66.2–89.8) | 76.7 (57.5–95.9) |
| ***Deep ICH location*** |  |  |  |  |  |  |  |
| Mean recovery time, months | 22.3 (19.4–23.2) | 13.6 (11.7–15.6) | 30.1 (25.7–34.5) | 53.0 (46.1–59.8) | 17.4 (15.4–19.4) | 46.6 (39.4–53.8) | 54.1 (44.0–64.1) |
| Median recovery time , months | 1.1 (0.8–1.4) | 0.7 (0.7–0.7) | 8.7 (0.9–16.5) | NA***** | 0.8 (0.7–0.9) | NA***** | NA***** |
| 3-month recovery rate, % | 59.9 (57.3–62.5) | 73.0 (70.3–75.7) | 35.7 (29.6–41.8) | 20.9 (14.8–26.9) | 65.4 (62.7–68.1) | 30.2 (23.1–37.3) | 16.7 (5.3–28.1) |
| 6-month recovery rate, % | 64.4 (61.8–66.9) | 76.5 (73.8–79.2) | 43.7 (37.4–50.0) | 24.8 (18.3–31.3) | 69.7 (67.2–72.2) | 35.7 (28.3–43.1) | 21.7 (9.2–34.2) |
| 1-year recovery rate, % | 68.6 (66.1–71.1) | 79.4 (76.9–81.9) | 51.4 (45.1–57.7) | 31.6 (24.5–38.8) | 74.1 (71.6–76.6) | 38.5 (30.9–46.1) | 26.8 (13.3–40.3) |
| 2-year recovery rate, % | 74.3 (71.8–76.7) | 84.1 (81.7–86.5) | 57.5 (51.0–64.0) | 41.2 (33.1–49.3) | 79.1 (76.7–81.5) | 49.3 (40.9–57.7) | 30.0 (15.7–44.3) |
| 3-year recovery rate, % | 78.4 (76.0–80.8) | 87.3 (84.9–89.7) | 64.3 (57.2–71.4) | 45.8 (37.1–54.4) | 83.6 (81.2–86.0) | 52.8 (44.0–61.6) | 30.0 (15.7–44.3) |
| Long-term recovery rate, % | 79.4 (77.0–81.9) | 88.4 (86.0–90.8) | 65.6 (58.3–72.9) | 45.8 (37.1–54.4) | 84.8 (82.4–87.2) | 52.8 (44.0–61.6) | 30.0 (15.7–44.3) |

Abbreviations: ICH, intracerebral hemorrhage; PHE, perihematomal edema.

*****The median time was not calculable for recovered patient proportion <50% in the corresponding group.

# Figure S1: Subgroup analyses of post-hemorrhagic motor recovery by ICH volume


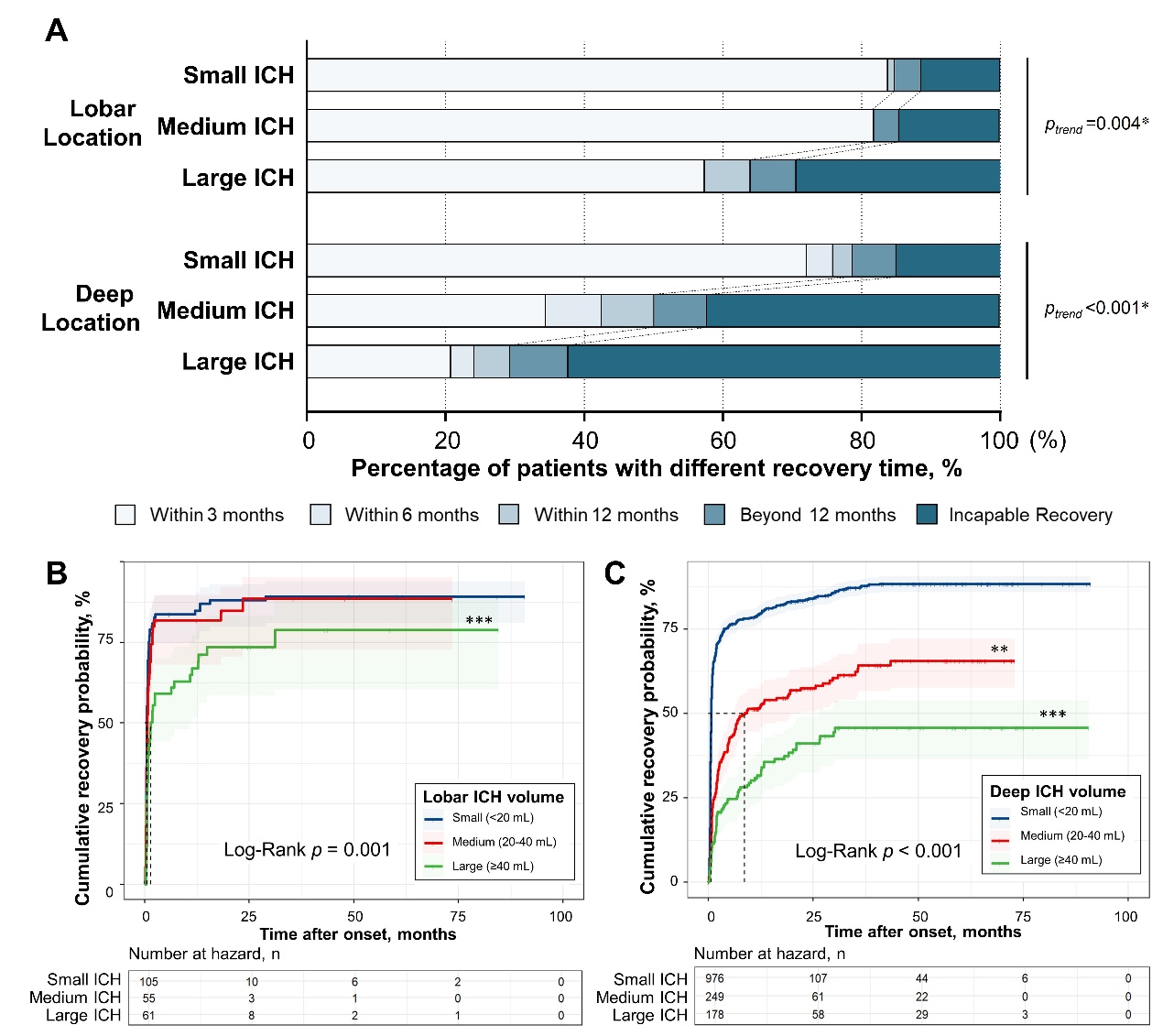


(A) Percentages of patients with different motor recovery times; (B) Kaplan-Meier (KM) event curves for lobar subgroup; (C) KM event curves for deep subgroup. ICH, intracerebral hemorrhage.

***** The *p-value* was calculated by Chi-square test for trend.

****** Adjusted *p-value* < 0.05 compared with small ICH group (<20mL), by BH method.

******* Adjusted *p-value* < 0.05 compared with both small and median ICH groups (<20mL and 20–40mL), by BH method.

# Figure S2: Subgroup analyses of post-hemorrhagic motor recovery by PHE volume


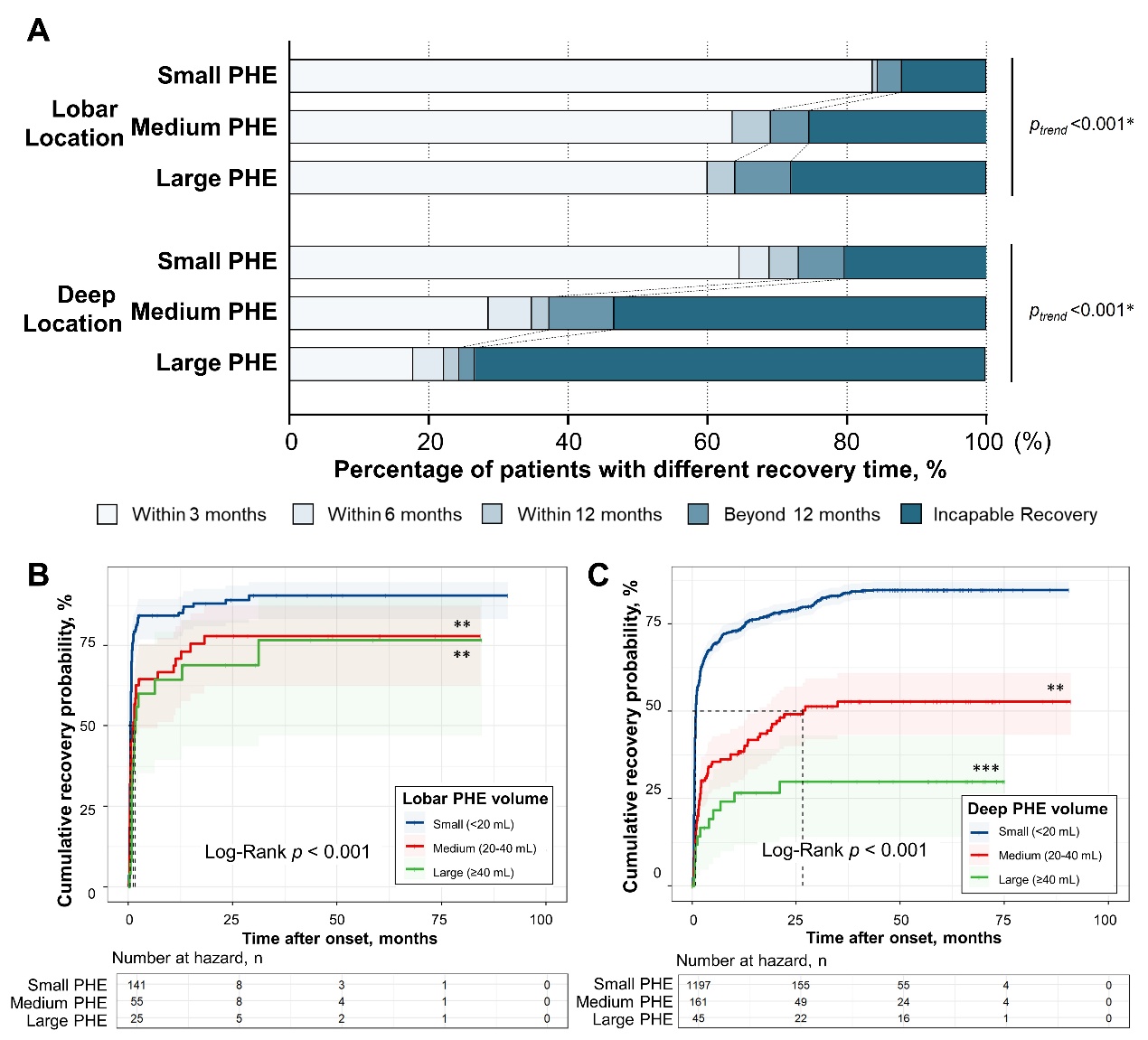


(A) Percentages of patients with different motor recovery times; (B) Kaplan-Meier (KM) event curves for lobar subgroup; (C) KM event curves for deep subgroup. ICH, intracerebral hemorrhage; PHE, perihematomal edema.

***** The *p-value* was calculated by Chi-square test for trend.

****** Adjusted *p-value* < 0.05 compared with small PHE group (<20mL), by BH method.

******* Adjusted *p-value* < 0.05 compared with both small and median PHE groups (<20mL and 20–40mL), by BH method.

# Table S2: Patient count of first motor recovery within post-stroke time by ICH volume

| **Recovery time** | **ICH volume** | | | | |
| --- | --- | --- | --- | --- | --- |
|  | **Small (< 20 mL) (n=922)** | **Medium (20–40 mL) (n=191)** | **Large (≥ 40 mL) (n=110)** | ***χ^2^*** | ***p_trend_**** |
| 3-months | 796 (86.3) | 132 (69.1) | 71 (64.5) | 71.33 | < 0.001 |
| 6-months | 33 (3.6) | 19 (9.9) | 6 (5.5) |  |  |
| 1-years | 26 (2.8) | 18 (9.4) | 14 (12.7) |  |  |
| 2-years | 41 (4.4) | 13 (6.8) | 14 (12.7) |  |  |
| 3-years | 21 (2.3) | 8 (4.2) | 5 (4.5) |  |  |
| 4-years | 5 (0.5) | 1 (0.5) | 0 |  |  |

Abbreviations: ICH, intracerebral hemorrhage.

***** The *P value* was calculated by *Chi-square* test for trend.

# Table S3: Patient count of first motor recovery within post-stroke time by PHE volume

| **Recovery time** | **PHE volume** | | | | |
| --- | --- | --- | --- | --- | --- |
|  | **Small (< 20 mL) (n=1077)** | **Medium (20–40 mL) (n=116)** | **Large (≥ 40 mL) (n=30)** | ***χ^2^*** | ***p_trend_**** |
| 3-months | 894 (83.0) | 83 (71.6) | 22 (73.3) | 18.30 | 0.03 |
| 6-months | 48 (4.5) | 8 (6.9) | 2 (6.7) |  |  |
| 1-years | 48 (4.5) | 7 (6.0) | 3 (10.0) |  |  |
| 2-years | 51 (4.7) | 15 (12.9) | 2 (6.7) |  |  |
| 3-years | 30 (2.8) | 3 (2.6) | 1 (3.3) |  |  |
| 4-years | 6 (0.6) | 0 | 0 |  |  |

Abbreviations: PHE, perihematomal edema.

***** The *P value* was calculated by *Chi-square* test for trend.

# Table S4: Patient count of first motor recovery within post-stroke time by ICH locations

| **Recovery time** | **ICH location** | | | |
| --- | --- | --- | --- | --- |
|  | **Lobar ICH (n=183)** | **Deep ICH (n=1040)** | ***χ^2^*** | ***p_trend_**** |
| 3-months | 168 (91.8) | 831 (79.9) | 8.55 | 0.004 |
| 6-months | 0 | 58 (5.6) |  |  |
| 1-years | 4 (2.2) | 54 (5.2) |  |  |
| 2-years | 9 (4.9) | 59 (5.7) |  |  |
| 3-years | 2 (1.1) | 32 (3.1) |  |  |
| 4-years | 0 | 6 (0.6) |  |  |

Abbreviations: ICH, intracerebral hemorrhage.

***** The *P value* was calculated by *Chi-square* test for trend.

#

# Table S5: Clinical characteristics and Cox proportional hazards regression models by 1-year motor outcomes

| **Characteristics** | **Entire analyzed population (n=1624)** | **Recovered group (n=1118)** | **Unrecovered group (n=506)** | ***p*-value** | **Univariate Cox regression** | |
| --- | --- | --- | --- | --- | --- | --- |
|  |  |  |  |  | **HR (95% CI)** | ***p*-value** |
| Age, mean (SD), years | 60.7 (12.7) | 58.8 (12.4) | 65.1 (12.5) | < 0.001 | 0.98 (0.98–0.99) | < 0.001 |
| Onset/LSW to baseline CT time, median (IQR), hours | 4.5 (2.2–11.0) | 4.5 (2.2–11.7) | 4.5 (2.2–9.4) | 0.18 | 1.01 (1.00–1.01) | 0.007 |
| Admission to referral hospitals | 1275 (78.5) | 846 (75.7) | 429 (84.8) | < 0.001 | 0.76 (0.66–0.87) | < 0.001 |
| Sex |  |  |  |  |  |  |
| Male | 1091 (67.2) | 738 (66.0) | 353 (69.8) | 0.14 | Reference | - |
| Female | 533 (32.8) | 380 (34.0) | 153 (30.2) |  | 1.09 (0.97–1.24) | 0.15 |
| Smoking |  |  |  |  |  |  |
| Never | 1289 (79.6) | 878 (78.5) | 411 (81.9) | 0.29 | Reference | - |
| Occasionally | 29 (1.8) | 21 (1.9) | 8 (1.6) |  | 0.96 (0.62–1.48) | 0.85 |
| Regular | 253 (15.6) | 180 (16.1) | 73 (14.5) |  | 1.12 (0.96–1.32) | 0.16 |
| Cessation | 49 (3.0) | 39 (3.5) | 10 (2.0) |  | 1.29 (0.93–1.77) | 0.13 |
| Drinking |  |  |  |  |  |  |
| Never | 1302 (80.4) | 885 (79.2) | 417 (82.9) | 0.32 | Reference | - |
| Occasionally | 116 (7.2) | 83 (7.4) | 33 (6.6) |  | 1.12 (0.90–1.41) | 0.31 |
| Regular | 187 (11.5) | 139 (12.4) | 48 (9.5) |  | 1.15 (0.96–1.37) | 0.13 |
| Cessation | 15 (0.9) | 10 (0.9) | 5 (1.0) |  | 1.12 (0.60–2.09) | 0.72 |
| Diabetes | 179 (11.0) | 117 (10.5) | 62 (12.3) | 0.30 | 0.90 (0.75–1.09) | 0.29 |
| Hypertension | 1139 (70.6) | 785 (70.5) | 354 (70.9) | 0.86 | 0.99 (0.87–1.12) | 0.84 |
| Premorbid mRS score |  |  |  |  |  |  |
| 0 | 1464 (90.2) | 1026 (91.8) | 438 (86.7) | 0.002 | Reference | - |
| 1 | 148 (9.1) | 88 (7.9) | 60 (11.9) |  | 0.76 (0.61–0.95) | 0.02 |
| 2 | 11 (0.7) | 4 (0.4) | 7 (1.4) |  | 0.48 (0.18–1.27) | 0.14 |
| Premorbid medication |  |  |  |  |  |  |
| Antiplatelet drugs | 44 (2.7) | 19 (1.7) | 25 (5.0) | < 0.001 | 0.48 (0.30–0.75) | 0.001 |
| Anticoagulant drugs | 12 (0.7) | 7 (0.6) | 5 (1.0) | 0.63 | 0.75 (0.36–1.59) | 0.46 |
| GCS score, point | 13 (11–15) | 14 (12–15) | 11 (8–13) | < 0.001 | 1.24 (1.21–1.28) | < 0.001 |
| 13–15 | 1004 (61.8) | 821 (73.4) | 183 (36.2) | < 0.001 | Reference | - |
| 9–12 | 406 (25.0) | 227 (20.3) | 179 (35.4) |  | 0.42 (0.36–0.49) | < 0.001 |
| 3–8 | 214 (13.2) | 70 (6.3) | 144 (28.5) |  | 0.20 (0.16–0.26) | < 0.001 |
| ICH location |  |  |  |  |  |  |
| Lobar | 221 (13.6) | 173 (15.5) | 48 (9.5) | 0.001 | Reference | - |
| Deep | 1403 (86.4) | 945 (84.5) | 458 (90.5) |  | 0.70 (0.59–0.82) | < 0.001 |
| Intraventricular hemorrhage | 546 (33.6) | 300 (26.8) | 246 (48.6) | < 0.001 | 0.54 (0.47–0.61) | < 0.001 |
| Surgical treatment | 411 (25.3) | 200 (17.9) | 211 (41.7) | < 0.001 | 0.42 (0.36–0.49) | < 0.001 |
| ICH volume, median (IQR), mL | 12.1 (5.2–27.4) | 9.3 (4.3–18.9) | 23.6 (10.6–43.8) | < 0.001 | 0.97 (0.97–0.98) | < 0.001 |
| Small (< 20 mL) | 1081 (66.6) | 857 (76.7) | 224 (44.3) | < 0.001 | Reference | - |
| Medium (20–40 mL) | 304 (18.7) | 170 (15.2) | 134 (26.5) |  | 0.47 (0.40–0.55) | < 0.001 |
| Large (≥ 40 mL) | 239 (14.7) | 91 (8.1) | 148 (29.2) |  | 0.29 (0.23–0.36) | < 0.001 |
| PHE volume, median (IQR), mL | 7.0 (2.9–15.6) | 5.4 (2.3–11.5) | 12.1 (5.5–23.1) | < 0.001 | 0.97 (0.96–0.97) | < 0.001 |
| Small (< 20 mL) | 1338 (82.4) | 993 (88.8) | 345 (68.2) | < 0.001 | Reference | - |
| Medium (20–40 mL) | 216 (13.3) | 98 (8.8) | 118 (23.3) |  | 0.43 (0.35–0.53) | < 0.001 |
| Large (≥ 40 mL) | 70 (4.3) | 27 (2.4) | 43 (8.5) |  | 0.35 (0.24–0.52) | < 0.001 |
| Post-discharge rehabilitation |  |  |  |  |  |  |
| No received | 646 (39.8) | 519 (46.4) | 127 (25.2) | < 0.001 | Reference | - |
| Received | 547 (33.7) | 408 (36.5) | 139 (27.6) |  | 0.69 (0.61–0.79) | < 0.001 |
| Unknown | 429 (26.4) | 191 (17.1) | 238 (47.2) |  | 0.41 (0.34–0.48) | < 0.001 |

Abbreviations: HR, hazard ratio; SD, standard deviation; IQR, interquartile range; LSW, last seen well; CT, computed tomography; GCS, Glasgow Coma Scale; mRS, modified Rankin scale; ICH, intracerebral hemorrhage; PHE, perihematomal edema.

# Table S6: Multivariable Cox proportional hazards regression model by 1-year motor outcomes

| **Characteristics** | ***a*HR (95% CI)** | ***Statistics*** | ***p*-value** |
| --- | --- | --- | --- |
|  |  |  |  |
| Age, per year | 0.98 (0.97–0.98) | 68.78 | <0.001 |
| Onset/LSW to baseline CT time, per hour | 1.00 (1.00–1.01) | 1.38 | 0.24 |
| Admission to referral hospitals | 0.79 (0.68–0.90) | 11.58 | 0.001 |
| Premorbid mRS score |  |  |  |
| 0 | Reference | - | - |
| 1 | 0.81 (0.65–1.02) | 3.28 | 0.07 |
| 2 | 0.69 (0.26–1.85) | 0.55 | 0.46 |
| Antiplatelet drugs | 0.63 (0.40–1.00) | 3.78 | 0.05 |
| GCS score***** |  |  |  |
| 13–15 | Reference | - | - |
| 9–12 | 0.54 (0.47–0.63) | 59.46 | <0.001 |
| 3–8 | 0.40 (0.31–0.52) | 47.06 | <0.001 |
| Deep ICH location | 0.55 (0.46–0.65) | 46.06 | <0.001 |
| Intraventricular hemorrhage | 0.70 (0.61–0.81) | 24.79 | <0.001 |
| Surgical treatment | 0.89 (0.74–1.08) | 1.39 | 0.24 |
| ICH volume, per mL | 0.98 (0.97–0.99) | 43.92 | <0.001 |
| PHE volume, per mL | 0.99 (0.98–1.00) | 4.51 | 0.03 |
| Post-discharge rehabilitation |  |  |  |
| No received | Reference | - | - |
| Received | 0.81 (0.71–0.93) | 9.05 | 0.003 |
| Unknown | 0.64 (0.54–0.76) | 26.12 | <0.001 |

Abbreviations: *a*HR, adjusted hazard ratio; CI: confidence interval; LSW, last seen well; CT, computed tomography; mRS, modified Rankin scale; GCS, Glasgow coma scale; ICH, intracerebral hemorrhage; PHE, perihematomal edema.

*****GCS grade was reserved in the multivariate model instead of the continuous variable.

# Table S7: Outcome assessments in popular studies

| **Study** | **Published year** | **Sample size** | **Latest follow-up time** | **Principle neurological outcome** |
| --- | --- | --- | --- | --- |
| STICH | 2005 | 1033 | 6 months | Prognostic equation |
| INTERACT | 2008 | 393 | 3 months | mRS score (≤2 *vs*. 2) |
| FAST | 2011 | 821 | 3 months | mRS score (≤4 *vs*. >4) |
| INTERACT-II | 2013 | 2794 | 3 months | mRS score (≤2 *vs*. 2) |
| STICH-II | 2013 | 601 | 6 months | eGOS score |
| ATACH-II | 2016 | 1000 | 3 months | mRS score (≤3 *vs*. >3) |
| CLEAR-III | 2017 | 500 | 6 months | mRS score (≤3 *vs*. >3) |
| MISTIE-III | 2019 | 506 | 1 year | mRS score (≥5 *vs*. <5) |
| INTERACT-III | 2023 | 7036 | 6 months | mRS score shift |
| SWITCH | 2024 | 201 | 6 months | mRS score (≥5 *vs*. <5) |
| ENRICH | 2024 | 300 | 6 months | utility-weighted mRS score |

Abbreviations: mRS, modified Rankin scale; eGOS, extended Glasgow outcome scale.
